# Supplementary material for: Trust and Trade-Offs in Sharing Data for Precision Medicine: A National Survey of Singapore
Source: J Pers Med. 2021 Sep 16;11(9):921. doi: 10.3390/jpm11090921 (PMC8465970; doi:10.3390/jpm11090921)
Supplement: Supplementary file 1 [file jpm-11-00921-s001.zip › Supplementary Figures 1-8 & Tables 1-4 & 6_16.09.21.pdf]

Supplementary Figures

Supplementary Figure S1 (s1). Part-worth utilities of the attribute levels by gender from the ACBC analysis. The width of the boxes corresponds with 95% confidence intervals.

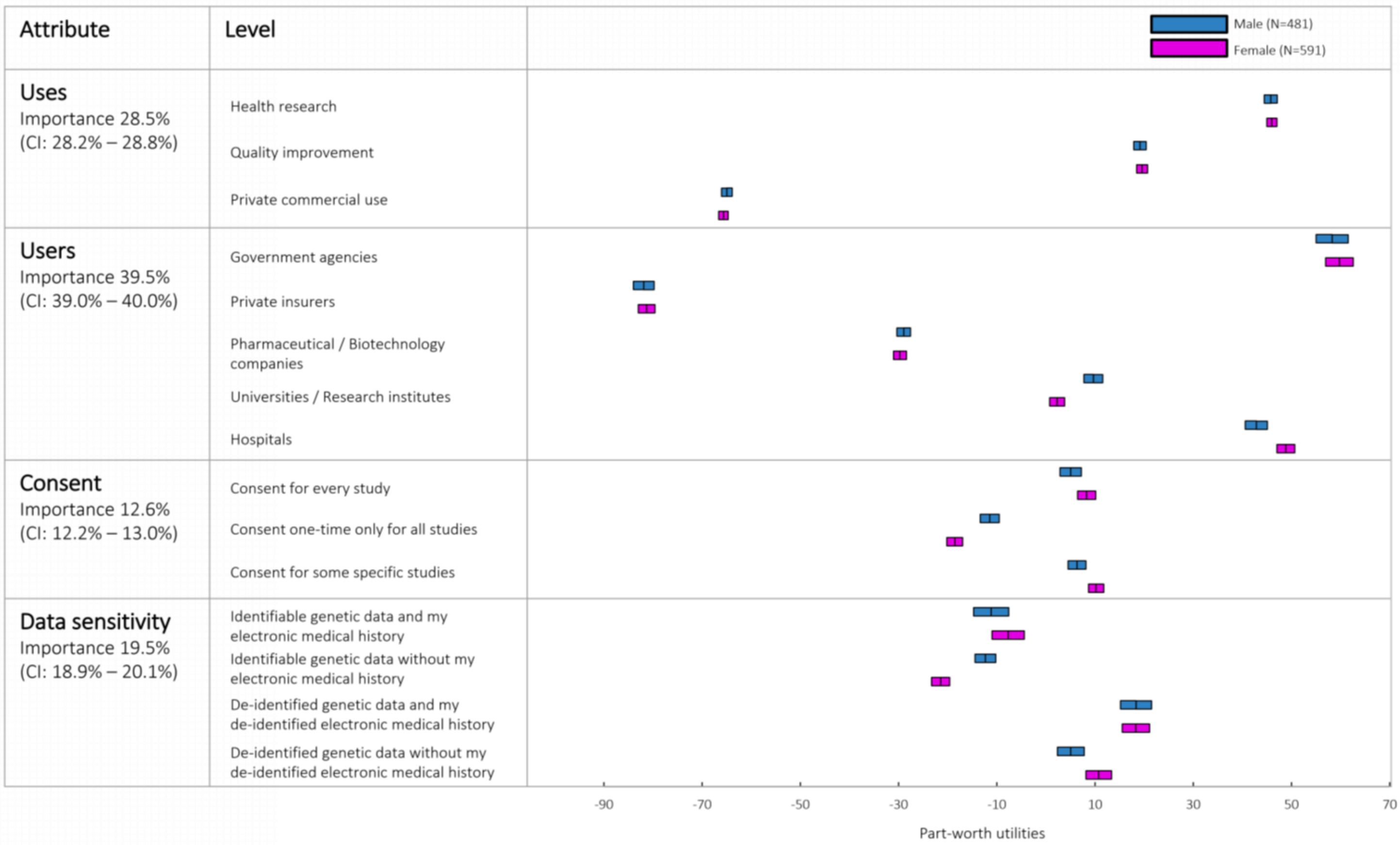

Supplementary Figure S2 (s2). Part-worth utilities of the attribute levels by ethnicity from the ACBC analysis. The width of the boxes corresponds with 95% confidence intervals.

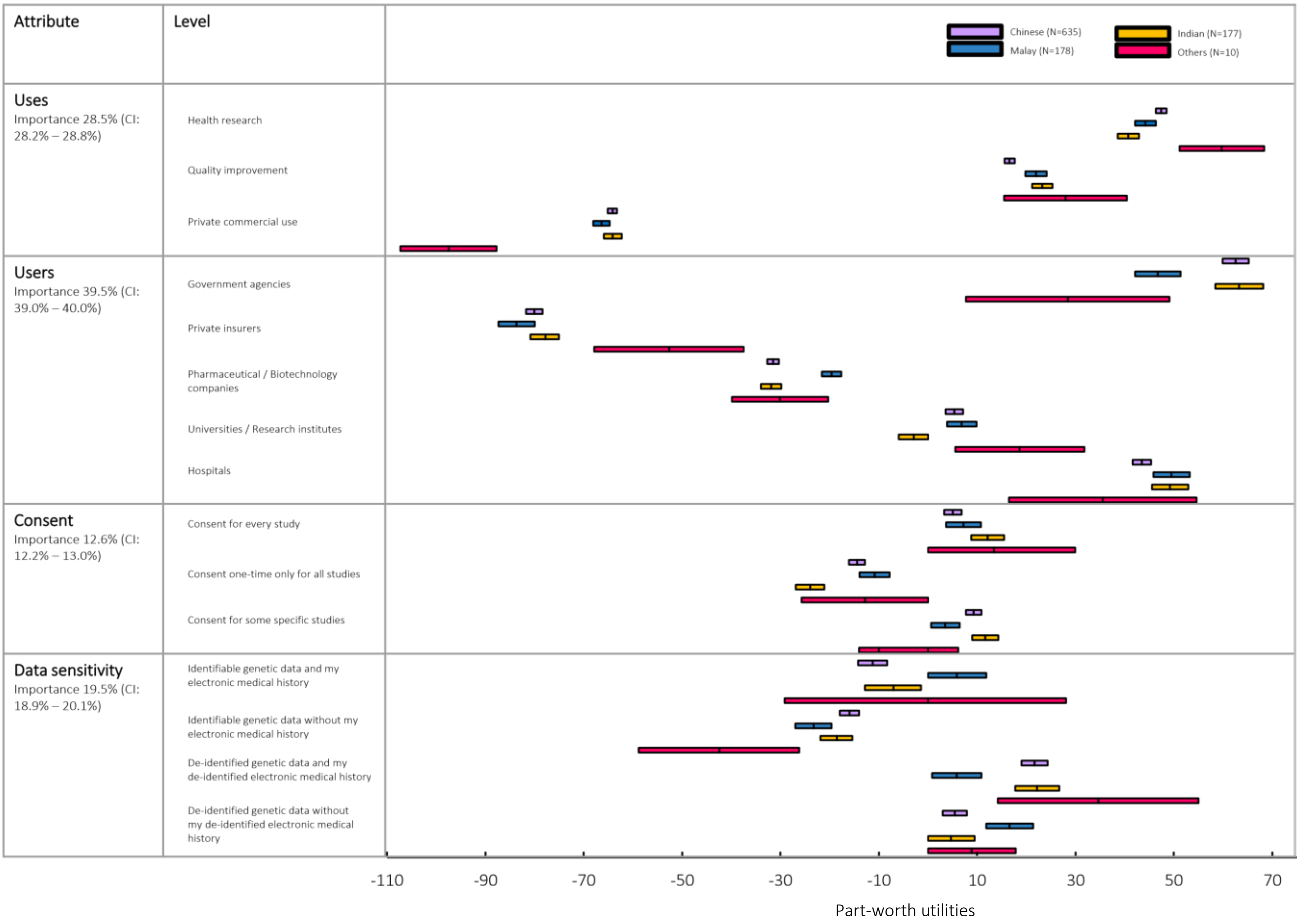

Supplementary Figure S3 (s3). Part-worth utilities of the attribute levels by religion from the ACBC analysis. The width of the boxes corresponds with 95% confidence intervals.

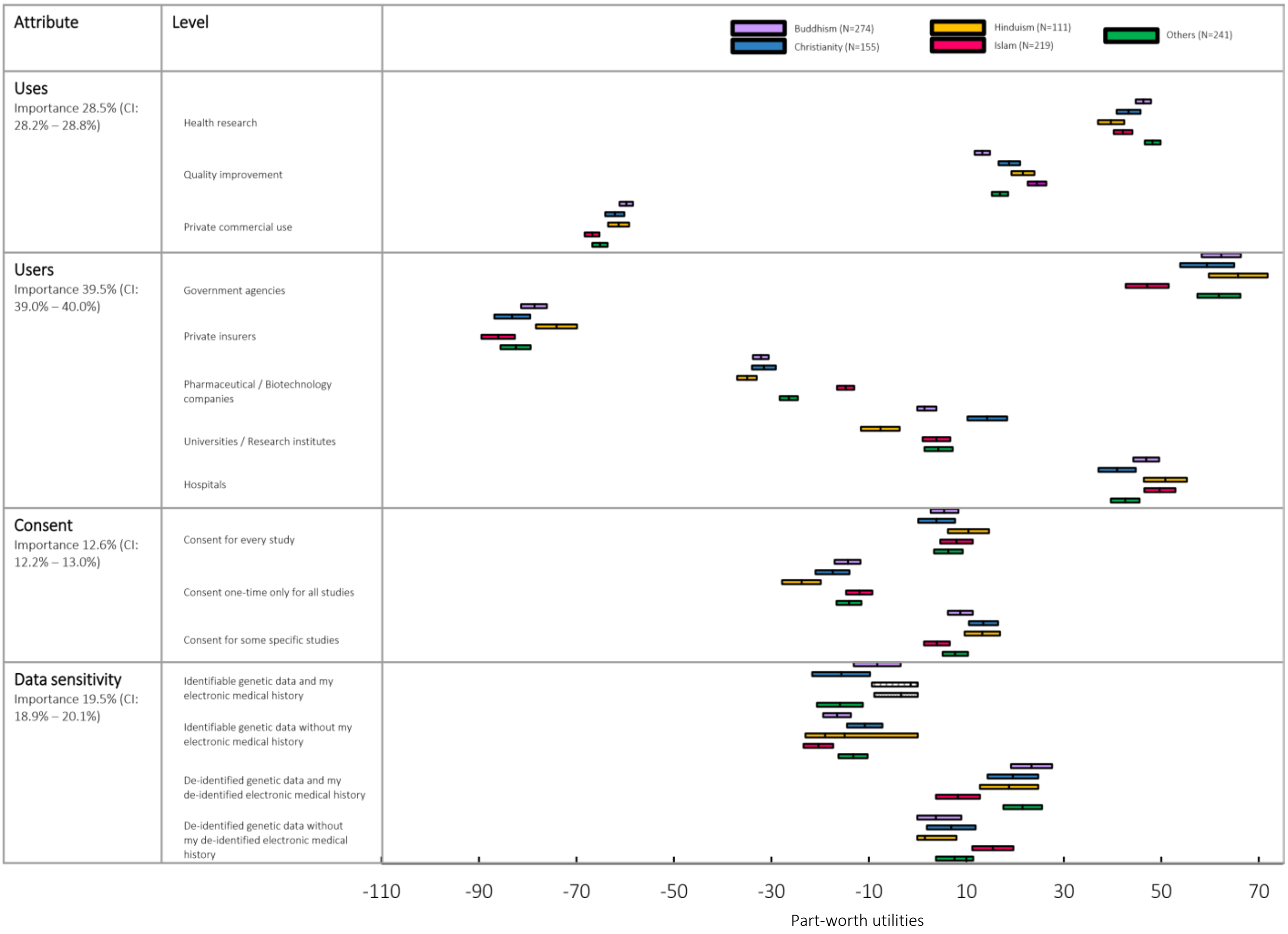

Supplementary Figure S4 (s4). Part-worth utilities of the attribute levels by age from the ACBC analysis. The width of the boxes corresponds with 95% confidence intervals.

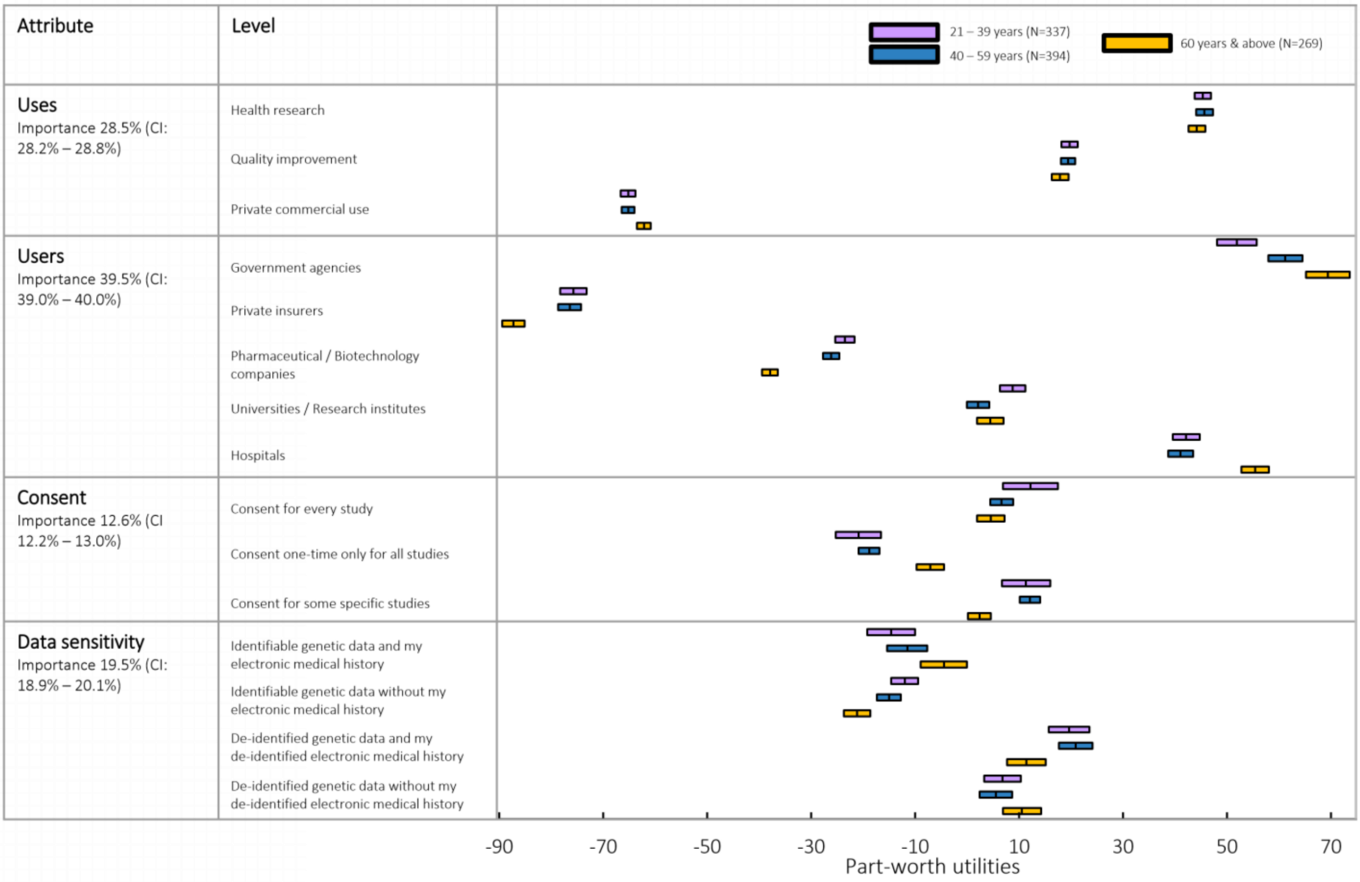

Supplementary Figure S5 (s5). Part-worth utilities of the attribute levels by education from the ACBC analysis. The width of the boxes corresponds with 95% confidence intervals.

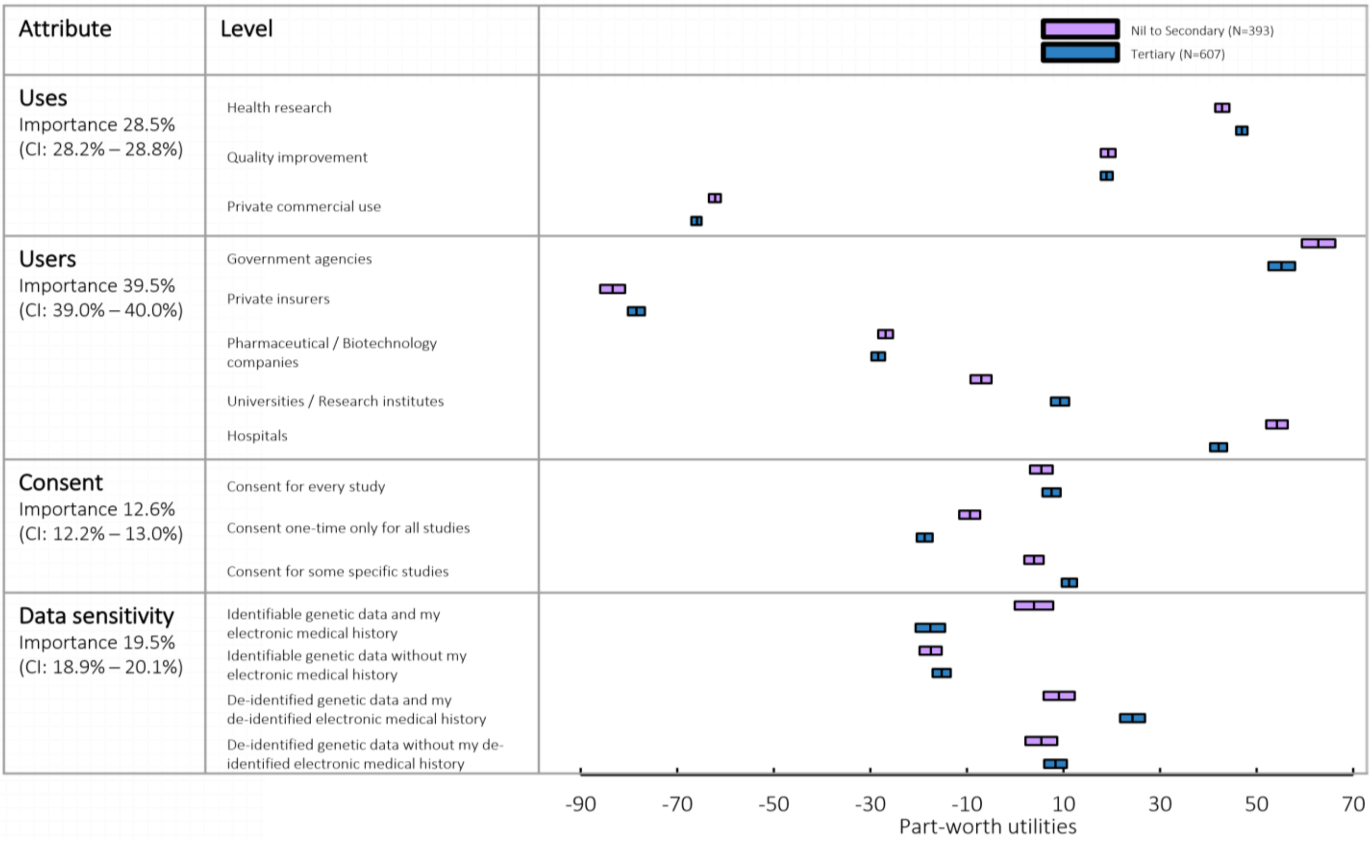

Supplementary Figure S6 (s6). Part-worth utilities of the attribute levels by self-reported health from the ACBC analysis. The width of the boxes corresponds with 95% confidence intervals.

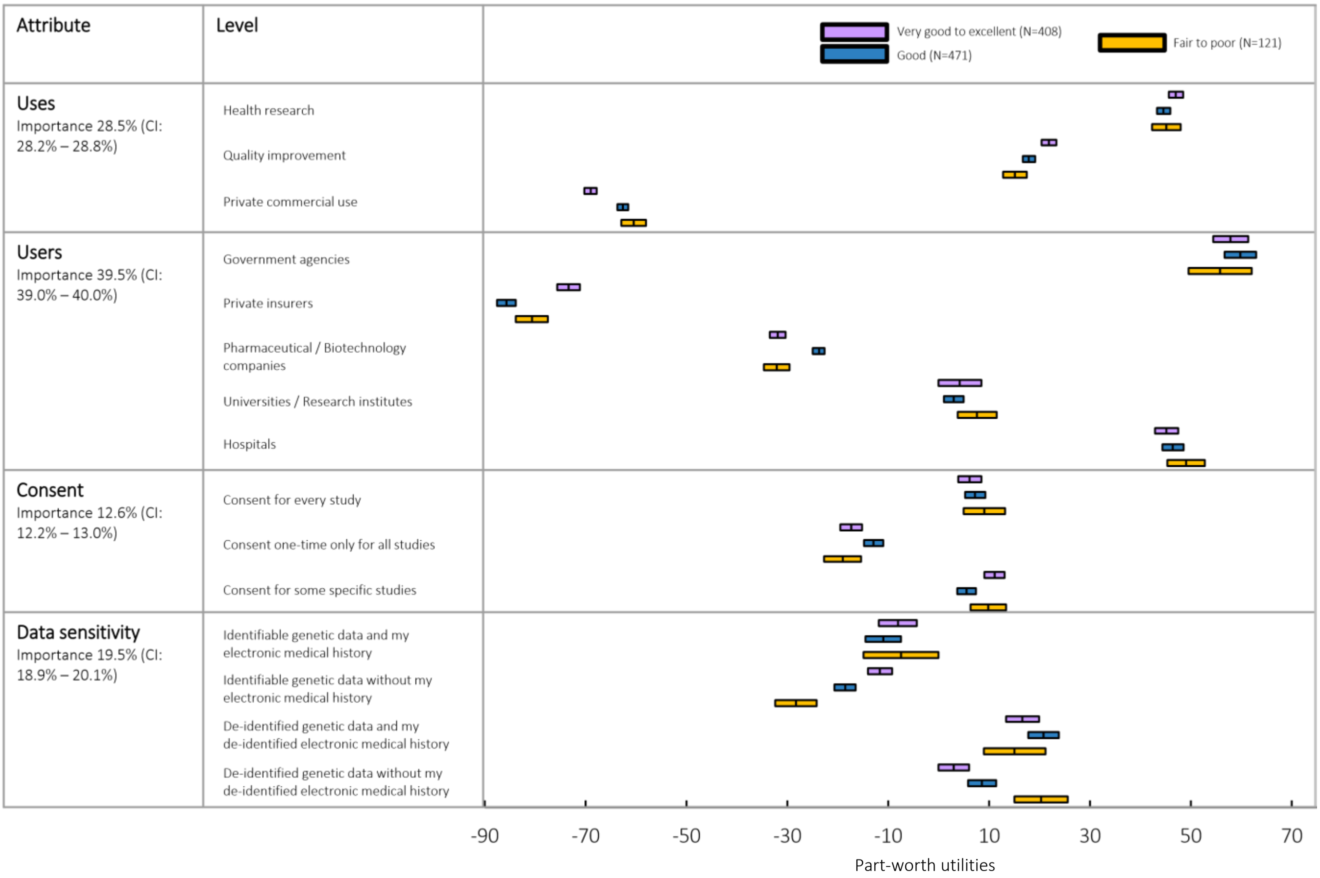

Supplementary Figure S7 (s7). Part-worth utilities of the attribute levels by housing type from the ACBC analysis. The width of the boxes corresponds with 95% confidence intervals.

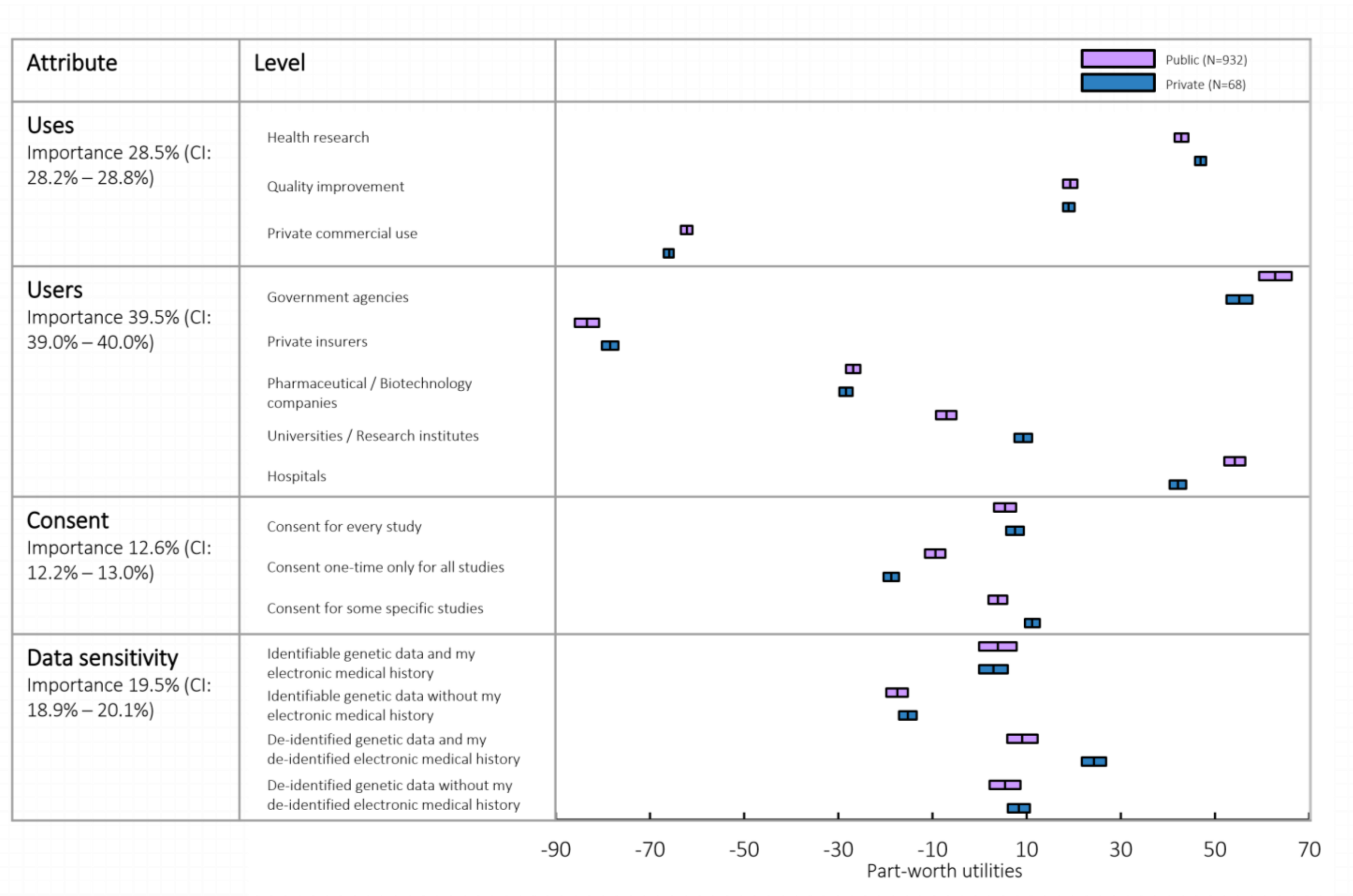

Supplementary Figure S8 (s8). Part-worth utilities of the attribute levels by income from the ACBC analysis. The width of the boxes corresponds with 95% confidence intervals.

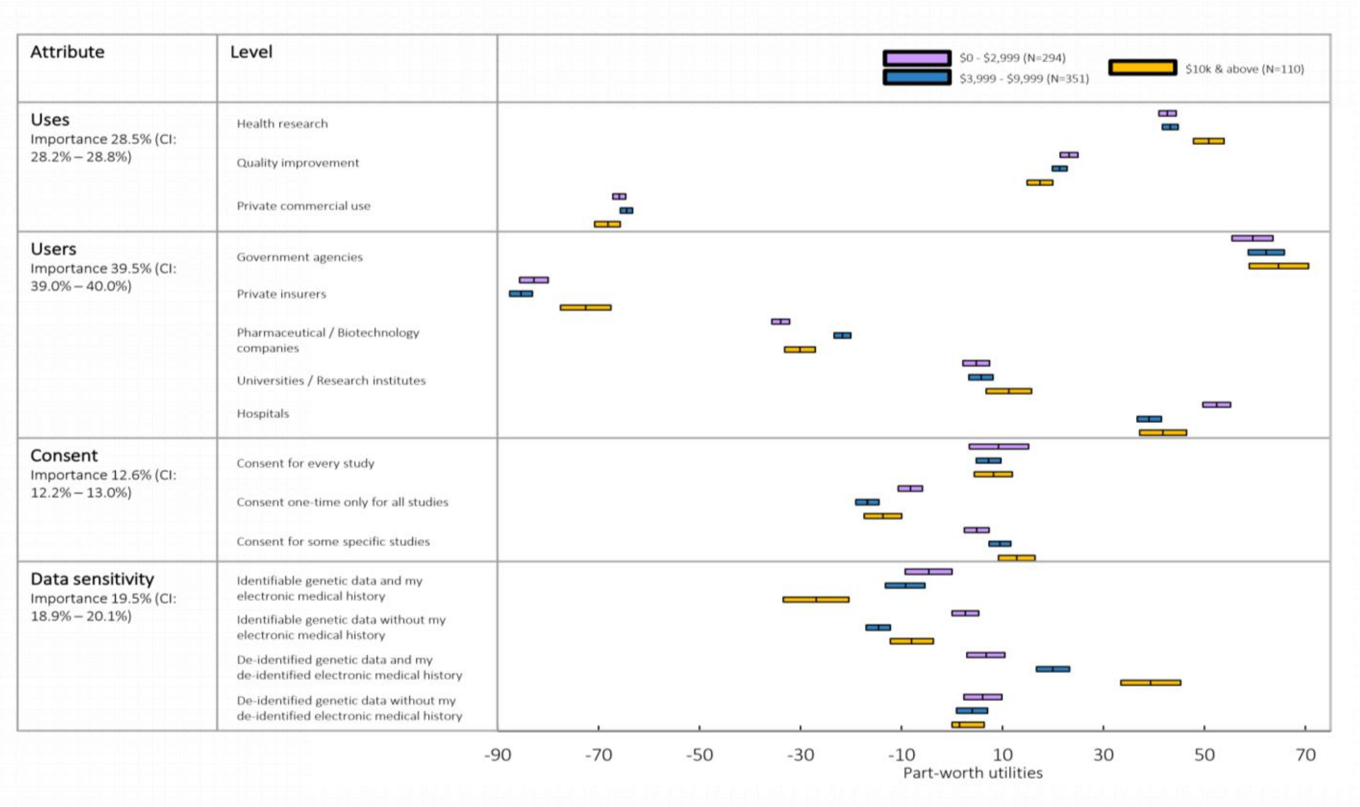

Supplementary Tables

Supplementary Table S1 (st1). Sampling frame provided by Department of Statistics Singapore (2019) for sample size N=1,000 by age, gender, and ethnicity, with oversampling of Malay and Indian respondents.

Age bracket	Chinese		Malay		Indian & Others	
	Male	Female	Male	Female	Male	Female
21-29	44	43	18	19	15	10
30-39	48	58	18	18	19	27
40-49	53	69	12	18	26	21
50-59	61	62	19	17	20	16
60-69	59	55	12	14	11	11
70 & above	36	47	5	8	5	6
**Total for each gender**	**301**	**334**	**84**	**94**	**96**	**91**
**Total for each race**	**635**		**178**		**187**	

Supplementary Table S2 (st2). Calculation of response rate based on American Association for Public Opinion Research (AAPOR) standards.[1]

Estimated proportion of doors knocked on that did not result in a (partial) interview	72%
Complete interviews (I)	1000
Partial interviews (P)	55
Number of doors knocked that did not result in a (partial) interview (NI)	(I + P) / (1 – 0.72) – I – P = 2713
Estimated proportion of NI who refused interviews	15%
Estimated proportion of NI who were determined to be not eligible	25%
Estimated proportion of NI who did not open the door	60%
Refused interviews (R)	0.15 * NI = 407
Unknown if the door knocked on contains an eligible household member (i.e. door was not opened.) (UH)	0.60 * NI = 1628
Estimated proportion of UH that are eligible (e)	1 - 0.25 * NI / (I + P + R) = 54%
Minimum response rate (RR1) (This counts all unopened doors as non-responses.)	I / (I + P + R + UH) = 32%
Maximum response rate (RR3) (This response rate can be considered conservative as it counts those that were not home as non-responses.)	I / (I + P + R + e*UH) = 43%

[1] American Association for Public Opinion Research. Final Dispositions of Case Codes and Outcome Rates for Surveys. Available at: https://www.aapor.org/AAPOR_Main/media/publications/Standard-Definitions20169theditionfinal.pdf Accessed June 4, 2021

Supplementary Table S3 (st3). Spearman's correlations between level of trust in institutions. Strong, moderate and weak correlations are defined as ≥0.50, 0.30–0.49 and 0.20–0.29, respectively.

	Hospitals	Medisave	Private genetic testing companies	Pharmaceutical companies	Private insurers	Facebook	IT Companies	Singapore medical societies	Government agencies	Ministry of Health	Universities / Research institutes	Overseas Universities
Pharmacies	0.549**	0.482**	0.396**	0.528**	0.393**	0.298**	0.322**	0.466**	0.440**	0.444**	0.481**	0.283**
Hospitals		0.845**	0.372**	0.407**	0.346**	0.095**	0.224**	0.731**	0.806**	0.835**	0.740**	0.309**
Medisave			0.345**	0.392**	0.354**	0.142**	0.248**	0.704**	0.830**	0.831**	0.720**	0.303**
Private genetic testing companies				0.860**	0.836**	0.489**	0.705**	0.309**	0.348**	0.376**	0.344**	0.790**
Pharmaceutical companies					0.830**	0.433**	0.669**	0.346**	0.396**	0.436**	0.378**	0.768**
Private insurers						0.511**	0.722**	0.296**	0.336**	0.352**	0.322**	0.766**
Facebook							0.821**	0.270**	0.158**	0.111**	0.295**	0.500**
IT Companies								0.307**	0.265**	0.247**	0.323**	0.761**
Singapore medical societies									0.776**	0.764**	0.829**	0.329**
Government agencies										0.932**	0.794**	0.333**
Ministry of Health											0.791**	0.334**
Universities / Research institutes												0.385**

**. Correlation is significantly different from zero at the 0.01 significance level (2-tailed).

Supplementary Table S4 (st4). Summary of results from simulations of what proportion of respondents would deem a specific scenario acceptable. The scenarios that were found to be acceptable by ≤49.9% of the respondents are shaded in grey. The simulations were weighted for ethnicity according to Singapore's demographics (i.e. 76.0% Chinese, 15.0% Indian, 7.5% Malay and 1.5% Others).

Scenario no.	Attribute level				Proportion of respondents who found scenario acceptable	Proportion of respondents who found scenario unacceptable
	Attribute level of uses	Attribute level of users	Attribute level of consent	Attribute level of data sensitivity		
1	Health research	Government agencies	Consent for every study	De-identified genetic data and my de-identified electronic medical history	87.8%	12.2%
2	Health research	Government agencies	Consent for every study	De-identified genetic data without my electronic medical history	87.0%	13.0%
3	Health research	Government agencies	Consent for every study	Identifiable genetic data and my electronic medical history	68.1%	31.9%
4	Health research	Government agencies	Consent for every study	Identifiable genetic data without my electronic medical history	69.0%	31.0%
5	Health research	Government agencies	Consent for some specific studies	De-identified genetic data and my de-identified electronic medical history	88.1%	11.9%
6	Health research	Government agencies	Consent for some specific studies	De-identified genetic data without my electronic medical history	87.2%	12.8%
7	Health research	Government agencies	Consent for some specific studies	Identifiable genetic data and my electronic medical history	70.1%	29.9%
8	Health research	Government agencies	Consent for some specific studies	Identifiable genetic data without my electronic medical history	70.0%	30.0%
9	Health research	Government agencies	Consent one-time only for all studies	De-identified genetic data and my de-identified electronic medical history	77.6%	22.4%
10	Health research	Government agencies	Consent one-time only for all studies	De-identified genetic data without my electronic medical history	75.8%	24.2%
11	Health research	Government agencies	Consent one-time only for all studies	Identifiable genetic data and my electronic medical history	59.5%	40.5%
12	Health research	Government agencies	Consent one-time only for all studies	Identifiable genetic data without my electronic medical history	58.5%	41.5%
13	Health research	Universities/Research institutes	Consent for every study	De-identified genetic data and my de-identified electronic medical history	65.8%	34.2%
14	Health research	Universities/Research institutes	Consent for every study	De-identified genetic data without my electronic medical history	61.7%	38.3%
15	Health research	Universities/Research institutes	Consent for every study	Identifiable genetic data and my electronic medical history	48.4%	51.6%
16	Health research	Universities/Research institutes	Consent for every study	Identifiable genetic data without my electronic medical history	46.0%	54.0%
17	Health research	Universities/Research institutes	Consent for some specific studies	De-identified genetic data and my de-identified electronic medical history	65.4%	34.6%
18	Health research	Universities/Research institutes	Consent for some specific studies	De-identified genetic data without my electronic medical history	61.3%	38.7%
19	Health research	Universities/Research institutes	Consent for some specific studies	Identifiable genetic data and my electronic medical history	48.6%	51.4%
20	Health research	Universities/Research institutes	Consent for some specific studies	Identifiable genetic data without my electronic medical history	46.2%	53.8%
21	Health research	Universities/Research institutes	Consent one-time only for all studies	De-identified genetic data and my de-identified electronic medical history	53.9%	46.1%
22	Health research	Universities/Research institutes	Consent one-time only for all studies	De-identified genetic data without my electronic medical history	47.8%	52.2%
23	Health research	Universities/Research institutes	Consent one-time only for all studies	Identifiable genetic data and my electronic medical history	40.5%	59.5%
24	Health research	Universities/Research institutes	Consent one-time only for all studies	Identifiable genetic data without my electronic medical history	37.6%	62.4%
25	Health research	Hospitals	Consent for every study	De-identified genetic data and my de-identified electronic medical history	85.2%	14.8%
26	Health research	Hospitals	Consent for every study	De-identified genetic data without my electronic medical history	84.0%	16.0%
27	Health research	Hospitals	Consent for every study	Identifiable genetic data and my electronic medical history	64.5%	35.5%
28	Health research	Hospitals	Consent for every study	Identifiable genetic data without my electronic medical history	65.3%	34.7%
29	Health research	Hospitals	Consent for some specific studies	De-identified genetic data and my de-identified electronic medical history	85.3%	14.7%

30	Health research	Hospitals	Consent for some specific studies	De-identified genetic data without my electronic medical history	83.7%	16.3%
31	Health research	Hospitals	Consent for some specific studies	Identifiable genetic data and my electronic medical history	66.0%	34.0%
32	Health research	Hospitals	Consent for some specific studies	Identifiable genetic data without my electronic medical history	66.0%	34.0%
33	Health research	Hospitals	Consent one-time only for all studies	De-identified genetic data and my de-identified electronic medical history	73.9%	26.1%
34	Health research	Hospitals	Consent one-time only for all studies	De-identified genetic data without my electronic medical history	70.9%	29.1%
35	Health research	Hospitals	Consent one-time only for all studies	Identifiable genetic data and my electronic medical history	55.3%	44.7%
36	Health research	Hospitals	Consent one-time only for all studies	Identifiable genetic data without my electronic medical history	53.6%	46.4%
37	Health research	Pharmaceutical / Biotechnology companies	Consent for every study	De-identified genetic data and my de-identified electronic medical history	48.5%	51.5%
38	Health research	Pharmaceutical / Biotechnology companies	Consent for every study	De-identified genetic data without my electronic medical history	40.8%	59.2%
39	Health research	Pharmaceutical / Biotechnology companies	Consent for every study	Identifiable genetic data and my electronic medical history	35.2%	64.8%
40	Health research	Pharmaceutical / Biotechnology companies	Consent for every study	Identifiable genetic data without my electronic medical history	31.1%	68.9%
41	Health research	Pharmaceutical / Biotechnology companies	Consent for some specific studies	De-identified genetic data and my de-identified electronic medical history	49.2%	50.8%
42	Health research	Pharmaceutical / Biotechnology companies	Consent for some specific studies	De-identified genetic data without my electronic medical history	42.1%	57.9%
43	Health research	Pharmaceutical / Biotechnology companies	Consent for some specific studies	Identifiable genetic data and my electronic medical history	35.3%	64.7%
44	Health research	Pharmaceutical / Biotechnology companies	Consent for some specific studies	Identifiable genetic data without my electronic medical history	31.6%	68.4%
45	Health research	Pharmaceutical / Biotechnology companies	Consent one-time only for all studies	De-identified genetic data and my de-identified electronic medical history	38.4%	61.6%
46	Health research	Pharmaceutical / Biotechnology companies	Consent one-time only for all studies	De-identified genetic data without my electronic medical history	30.5%	69.5%
47	Health research	Pharmaceutical / Biotechnology companies	Consent one-time only for all studies	Identifiable genetic data and my electronic medical history	29.5%	70.5%
48	Health research	Pharmaceutical / Biotechnology companies	Consent one-time only for all studies	Identifiable genetic data without my electronic medical history	26.1%	73.9%
49	Health research	Private insurers	Consent for every study	De-identified genetic data and my de-identified electronic medical history	24.9%	75.1%
50	Health research	Private insurers	Consent for every study	De-identified genetic data without my electronic medical history	17.2%	82.8%
51	Health research	Private insurers	Consent for every study	Identifiable genetic data and my electronic medical history	20.5%	79.5%
52	Health research	Private insurers	Consent for every study	Identifiable genetic data without my electronic medical history	16.6%	83.4%
53	Health research	Private insurers	Consent for some specific studies	De-identified genetic data and my de-identified electronic medical history	26.9%	73.1%
54	Health research	Private insurers	Consent for some specific studies	De-identified genetic data without my electronic medical history	19.8%	80.2%
55	Health research	Private insurers	Consent for some specific studies	Identifiable genetic data and my electronic medical history	20.8%	79.2%
56	Health research	Private insurers	Consent for some specific studies	Identifiable genetic data without my electronic medical history	17.6%	82.4%
57	Health research	Private insurers	Consent one-time only for all studies	De-identified genetic data and my de-identified electronic medical history	20.1%	79.9%

58	Health research	Private insurers	Consent one-time only for all studies	De-identified genetic data without my electronic medical history	13.8%	86.2%
59	Health research	Private insurers	Consent one-time only for all studies	Identifiable genetic data and my electronic medical history	18.2%	81.8%
60	Health research	Private insurers	Consent one-time only for all studies	Identifiable genetic data without my electronic medical history	15.3%	84.7%
61	Quality improvement	Government agencies	Consent for every study	De-identified genetic data and my de-identified electronic medical history	79.5%	20.5%
62	Quality improvement	Government agencies	Consent for every study	De-identified genetic data without my electronic medical history	76.4%	23.6%
63	Quality improvement	Government agencies	Consent for every study	Identifiable genetic data and my electronic medical history	57.7%	42.3%
64	Quality improvement	Government agencies	Consent for every study	Identifiable genetic data without my electronic medical history	57.0%	43.0%
65	Quality improvement	Government agencies	Consent for some specific studies	De-identified genetic data and my de-identified electronic medical history	79.1%	20.9%
66	Quality improvement	Government agencies	Consent for some specific studies	De-identified genetic data without my electronic medical history	76.0%	24.0%
67	Quality improvement	Government agencies	Consent for some specific studies	Identifiable genetic data and my electronic medical history	59.2%	40.8%
68	Quality improvement	Government agencies	Consent for some specific studies	Identifiable genetic data without my electronic medical history	57.9%	42.1%
69	Quality improvement	Government agencies	Consent one-time only for all studies	De-identified genetic data and my de-identified electronic medical history	66.7%	33.3%
70	Quality improvement	Government agencies	Consent one-time only for all studies	De-identified genetic data without my electronic medical history	62.3%	37.7%
71	Quality improvement	Government agencies	Consent one-time only for all studies	Identifiable genetic data and my electronic medical history	49.8%	50.2%
72	Quality improvement	Government agencies	Consent one-time only for all studies	Identifiable genetic data without my electronic medical history	47.8%	52.2%
73	Quality improvement	Universities/Research institutes	Consent for every study	De-identified genetic data and my de-identified electronic medical history	51.1%	48.9%
74	Quality improvement	Universities/Research institutes	Consent for every study	De-identified genetic data without my electronic medical history	43.7%	56.3%
75	Quality improvement	Universities/Research institutes	Consent for every study	Identifiable genetic data and my electronic medical history	37.0%	63.0%
76	Quality improvement	Universities/Research institutes	Consent for every study	Identifiable genetic data without my electronic medical history	33.2%	66.8%
77	Quality improvement	Universities/Research institutes	Consent for some specific studies	De-identified genetic data and my de-identified electronic medical history	51.4%	48.6%
78	Quality improvement	Universities/Research institutes	Consent for some specific studies	De-identified genetic data without my electronic medical history	44.6%	55.4%
79	Quality improvement	Universities/Research institutes	Consent for some specific studies	Identifiable genetic data and my electronic medical history	37.3%	62.7%
80	Quality improvement	Universities/Research institutes	Consent for some specific studies	Identifiable genetic data without my electronic medical history	33.9%	66.1%
81	Quality improvement	Universities/Research institutes	Consent one-time only for all studies	De-identified genetic data and my de-identified electronic medical history	40.4%	59.6%
82	Quality improvement	Universities/Research institutes	Consent one-time only for all studies	De-identified genetic data without my electronic medical history	32.9%	67.1%
83	Quality improvement	Universities/Research institutes	Consent one-time only for all studies	Identifiable genetic data and my electronic medical history	31.2%	68.8%
84	Quality improvement	Universities/Research institutes	Consent one-time only for all studies	Identifiable genetic data without my electronic medical history	27.7%	72.3%
85	Quality improvement	Hospitals	Consent for every study	De-identified genetic data and my de-identified electronic medical history	74.6%	25.4%
86	Quality improvement	Hospitals	Consent for every study	De-identified genetic data without my electronic medical history	70.0%	30.0%
87	Quality improvement	Hospitals	Consent for every study	Identifiable genetic data and my electronic medical history	53.3%	46.7%
88	Quality improvement	Hospitals	Consent for every study	Identifiable genetic data without my electronic medical history	51.6%	48.4%
89	Quality improvement	Hospitals	Consent for some specific studies	De-identified genetic data and my de-identified electronic medical history	74.2%	25.8%
90	Quality improvement	Hospitals	Consent for some specific studies	De-identified genetic data without my electronic medical history	69.6%	30.4%
91	Quality improvement	Hospitals	Consent for some specific studies	Identifiable genetic data and my electronic medical history	54.0%	46.0%

92	Quality improvement	Hospitals	Consent for some specific studies	Identifiable genetic data without my electronic medical history	52.1%	47.9%
93	Quality improvement	Hospitals	Consent one-time only for all studies	De-identified genetic data and my de-identified electronic medical history	61.1%	38.9%
94	Quality improvement	Hospitals	Consent one-time only for all studies	De-identified genetic data without my electronic medical history	55.1%	44.9%
95	Quality improvement	Hospitals	Consent one-time only for all studies	Identifiable genetic data and my electronic medical history	44.5%	55.5%
96	Quality improvement	Hospitals	Consent one-time only for all studies	Identifiable genetic data without my electronic medical history	41.9%	58.1%
97	Quality improvement	Pharmaceutical / Biotechnology companies	Consent for every study	De-identified genetic data and my de-identified electronic medical history	33.6%	66.4%
98	Quality improvement	Pharmaceutical / Biotechnology companies	Consent for every study	De-identified genetic data without my electronic medical history	25.5%	74.5%
99	Quality improvement	Pharmaceutical / Biotechnology companies	Consent for every study	Identifiable genetic data and my electronic medical history	26.0%	74.0%
100	Quality improvement	Pharmaceutical / Biotechnology companies	Consent for every study	Identifiable genetic data without my electronic medical history	21.5%	78.5%
101	Quality improvement	Pharmaceutical / Biotechnology companies	Consent for some specific studies	De-identified genetic data and my de-identified electronic medical history	35.3%	64.7%
102	Quality improvement	Pharmaceutical / Biotechnology companies	Consent for some specific studies	De-identified genetic data without my electronic medical history	28.0%	72.0%
103	Quality improvement	Pharmaceutical / Biotechnology companies	Consent for some specific studies	Identifiable genetic data and my electronic medical history	26.0%	74.0%
104	Quality improvement	Pharmaceutical / Biotechnology companies	Consent for some specific studies	Identifiable genetic data without my electronic medical history	22.3%	77.7%
105	Quality improvement	Pharmaceutical / Biotechnology companies	Consent one-time only for all studies	De-identified genetic data and my de-identified electronic medical history	26.5%	73.5%
106	Quality improvement	Pharmaceutical / Biotechnology companies	Consent one-time only for all studies	De-identified genetic data without my electronic medical history	19.5%	80.5%
107	Quality improvement	Pharmaceutical / Biotechnology companies	Consent one-time only for all studies	Identifiable genetic data and my electronic medical history	22.4%	77.6%
108	Quality improvement	Pharmaceutical / Biotechnology companies	Consent one-time only for all studies	Identifiable genetic data without my electronic medical history	19.0%	81.0%
109	Quality improvement	Private insurers	Consent for every study	De-identified genetic data and my de-identified electronic medical history	15.6%	84.4%
110	Quality improvement	Private insurers	Consent for every study	De-identified genetic data without my electronic medical history	10.1%	89.9%
111	Quality improvement	Private insurers	Consent for every study	Identifiable genetic data and my electronic medical history	14.9%	85.1%
112	Quality improvement	Private insurers	Consent for every study	Identifiable genetic data without my electronic medical history	11.4%	88.6%
113	Quality improvement	Private insurers	Consent for some specific studies	De-identified genetic data and my de-identified electronic medical history	17.9%	82.1%
114	Quality improvement	Private insurers	Consent for some specific studies	De-identified genetic data without my electronic medical history	12.8%	87.2%
115	Quality improvement	Private insurers	Consent for some specific studies	Identifiable genetic data and my electronic medical history	15.0%	85.0%
116	Quality improvement	Private insurers	Consent for some specific studies	Identifiable genetic data without my electronic medical history	12.3%	87.7%
117	Quality improvement	Private insurers	Consent one-time only for all studies	De-identified genetic data and my de-identified electronic medical history	13.2%	86.8%
118	Quality improvement	Private insurers	Consent one-time only for all studies	De-identified genetic data without my electronic medical history	8.7%	91.3%
119	Quality improvement	Private insurers	Consent one-time only for all studies	Identifiable genetic data and my electronic medical history	13.6%	86.4%

120	Quality improvement	Private insurers	Consent one-time only for all studies	Identifiable genetic data without my electronic medical history	11.1%	88.9%
121	Private commercial use	Government agencies	Consent for every study	De-identified genetic data and my de-identified electronic medical history	37.1%	62.9%
122	Private commercial use	Government agencies	Consent for every study	De-identified genetic data without my electronic medical history	28.9%	71.1%
123	Private commercial use	Government agencies	Consent for every study	Identifiable genetic data and my electronic medical history	28.7%	71.3%
124	Private commercial use	Government agencies	Consent for every study	Identifiable genetic data without my electronic medical history	24.3%	75.7%
125	Private commercial use	Government agencies	Consent for some specific studies	De-identified genetic data and my de-identified electronic medical history	38.5%	61.5%
126	Private commercial use	Government agencies	Consent for some specific studies	De-identified genetic data without my electronic medical history	30.8%	69.2%
127	Private commercial use	Government agencies	Consent for some specific studies	Identifiable genetic data and my electronic medical history	28.5%	71.5%
128	Private commercial use	Government agencies	Consent for some specific studies	Identifiable genetic data without my electronic medical history	24.9%	75.1%
129	Private commercial use	Government agencies	Consent one-time only for all studies	De-identified genetic data and my de-identified electronic medical history	29.3%	70.7%
130	Private commercial use	Government agencies	Consent one-time only for all studies	De-identified genetic data without my electronic medical history	22.2%	77.8%
131	Private commercial use	Government agencies	Consent one-time only for all studies	Identifiable genetic data and my electronic medical history	24.6%	75.4%
132	Private commercial use	Government agencies	Consent one-time only for all studies	Identifiable genetic data without my electronic medical history	21.3%	78.7%
133	Private commercial use	Universities/Research institutes	Consent for every study	De-identified genetic data and my de-identified electronic medical history	15.4%	84.6%
134	Private commercial use	Universities/Research institutes	Consent for every study	De-identified genetic data without my electronic medical history	9.0%	91.0%
135	Private commercial use	Universities/Research institutes	Consent for every study	Identifiable genetic data and my electronic medical history	13.7%	86.3%
136	Private commercial use	Universities/Research institutes	Consent for every study	Identifiable genetic data without my electronic medical history	9.6%	90.4%
137	Private commercial use	Universities/Research institutes	Consent for some specific studies	De-identified genetic data and my de-identified electronic medical history	17.5%	82.5%
138	Private commercial use	Universities/Research institutes	Consent for some specific studies	De-identified genetic data without my electronic medical history	11.7%	88.3%
139	Private commercial use	Universities/Research institutes	Consent for some specific studies	Identifiable genetic data and my electronic medical history	13.6%	86.4%
140	Private commercial use	Universities/Research institutes	Consent for some specific studies	Identifiable genetic data without my electronic medical history	10.5%	89.5%
141	Private commercial use	Universities/Research institutes	Consent one-time only for all studies	De-identified genetic data and my de-identified electronic medical history	12.7%	87.3%
142	Private commercial use	Universities/Research institutes	Consent one-time only for all studies	De-identified genetic data without my electronic medical history	7.6%	92.4%
143	Private commercial use	Universities/Research institutes	Consent one-time only for all studies	Identifiable genetic data and my electronic medical history	12.3%	87.7%
144	Private commercial use	Universities/Research institutes	Consent one-time only for all studies	Identifiable genetic data without my electronic medical history	9.7%	90.3%
145	Private commercial use	Hospitals	Consent for every study	De-identified genetic data and my de-identified electronic medical history	28.7%	71.3%
146	Private commercial use	Hospitals	Consent for every study	De-identified genetic data without my electronic medical history	20.2%	79.8%
147	Private commercial use	Hospitals	Consent for every study	Identifiable genetic data and my electronic medical history	23.0%	77.0%
148	Private commercial use	Hospitals	Consent for every study	Identifiable genetic data without my electronic medical history	17.9%	82.1%
149	Private commercial use	Hospitals	Consent for some specific studies	De-identified genetic data and my de-identified electronic medical history	30.4%	69.6%
150	Private commercial use	Hospitals	Consent for some specific studies	De-identified genetic data without my electronic medical history	22.5%	77.5%
151	Private commercial use	Hospitals	Consent for some specific studies	Identifiable genetic data and my electronic medical history	22.6%	77.4%
152	Private commercial use	Hospitals	Consent for some specific studies	Identifiable genetic data without my electronic medical history	18.3%	81.7%
153	Private commercial use	Hospitals	Consent one-time only for all studies	De-identified genetic data and my de-identified electronic medical history	21.9%	78.1%

154	Private commercial use	Hospitals	Consent one-time only for all studies	De-identified genetic data without my electronic medical history	15.1%	84.9%
155	Private commercial use	Hospitals	Consent one-time only for all studies	Identifiable genetic data and my electronic medical history	19.6%	80.4%
156	Private commercial use	Hospitals	Consent one-time only for all studies	Identifiable genetic data without my electronic medical history	15.9%	84.1%
157	Private commercial use	Pharmaceutical / Biotechnology companies	Consent for every study	De-identified genetic data and my de-identified electronic medical history	8.2%	91.8%
158	Private commercial use	Pharmaceutical / Biotechnology companies	Consent for every study	De-identified genetic data without my electronic medical history	4.2%	95.8%
159	Private commercial use	Pharmaceutical / Biotechnology companies	Consent for every study	Identifiable genetic data and my electronic medical history	8.6%	91.4%
160	Private commercial use	Pharmaceutical / Biotechnology companies	Consent for every study	Identifiable genetic data without my electronic medical history	5.4%	94.6%
161	Private commercial use	Pharmaceutical / Biotechnology companies	Consent for some specific studies	De-identified genetic data and my de-identified electronic medical history	9.7%	90.3%
162	Private commercial use	Pharmaceutical / Biotechnology companies	Consent for some specific studies	De-identified genetic data without my electronic medical history	5.7%	94.3%
163	Private commercial use	Pharmaceutical / Biotechnology companies	Consent for some specific studies	Identifiable genetic data and my electronic medical history	8.0%	92.0%
164	Private commercial use	Pharmaceutical / Biotechnology companies	Consent for some specific studies	Identifiable genetic data without my electronic medical history	5.8%	94.2%
165	Private commercial use	Pharmaceutical / Biotechnology companies	Consent one-time only for all studies	De-identified genetic data and my de-identified electronic medical history	7.0%	93.0%
166	Private commercial use	Pharmaceutical / Biotechnology companies	Consent one-time only for all studies	De-identified genetic data without my electronic medical history	3.6%	96.4%
167	Private commercial use	Pharmaceutical / Biotechnology companies	Consent one-time only for all studies	Identifiable genetic data and my electronic medical history	7.8%	92.2%
168	Private commercial use	Pharmaceutical / Biotechnology companies	Consent one-time only for all studies	Identifiable genetic data without my electronic medical history	5.7%	94.3%
169	Private commercial use	Private insurers	Consent for every study	De-identified genetic data and my de-identified electronic medical history	3.3%	96.7%
170	Private commercial use	Private insurers	Consent for every study	De-identified genetic data without my electronic medical history	1.9%	98.1%
171	Private commercial use	Private insurers	Consent for every study	Identifiable genetic data and my electronic medical history	5.0%	95.0%
172	Private commercial use	Private insurers	Consent for every study	Identifiable genetic data without my electronic medical history	3.2%	96.8%
173	Private commercial use	Private insurers	Consent for some specific studies	De-identified genetic data and my de-identified electronic medical history	4.3%	95.7%
174	Private commercial use	Private insurers	Consent for some specific studies	De-identified genetic data without my electronic medical history	2.6%	97.4%
175	Private commercial use	Private insurers	Consent for some specific studies	Identifiable genetic data and my electronic medical history	4.7%	95.3%
176	Private commercial use	Private insurers	Consent for some specific studies	Identifiable genetic data without my electronic medical history	3.4%	96.6%
177	Private commercial use	Private insurers	Consent one-time only for all studies	De-identified genetic data and my de-identified electronic medical history	3.3%	96.7%
178	Private commercial use	Private insurers	Consent one-time only for all studies	De-identified genetic data without my electronic medical history	1.8%	98.2%
179	Private commercial use	Private insurers	Consent one-time only for all studies	Identifiable genetic data and my electronic medical history	4.8%	95.2%
180	Private commercial use	Private insurers	Consent one-time only for all studies	Identifiable genetic data without my electronic medical history	3.5%	96.5%

Supplementary Table S6 (st6). Further details on the survey recruitment process.

Further details on the survey recruitment process
Twenty development guide plans (DGPs) with five primary sampling units (PSUs) Primary Sampling Units (stratified by dwelling type) in each, were selected. These 100 PSUs are representative of the dwelling-type distribution in Singapore and each of those had four add-on units selected using a one floor up or down approach to ensure the same house type was selected. One respondent was chosen from the selected household and screened according to the eligibility criteria, i.e., population quota breakdown by age, sex and race. In the event that the household was not eligible (outright refusal; no eligible respondent based on quota; no response after three attempts across different days and times), a replacement unit was sought to the right of the selected unit.
